# Liposomal iron dramatic effect on chronic kidney disease pediatric anemia: a randomized clinical trial

**DOI:** 10.1038/s41390-025-04418-x

**Published:** 2025-09-30

**Authors:** Sahar Kamal Hegazy, Mai Salah El-Din Koura, Mohamed Shokry Elharoun

**Affiliations:** 1 Faculty of Pharmacy, Tanta University, Tanta, Egypt; 2 Senior critical care clinical pharmacy, Menoufia University Hospitals, MSc in pharmacy, Faculty of Pharmacy, Tanta University, Shebin El-Kom, Egypt; 3 Faculty of Medicine, Menoufia University, Shebin El-Kom, Egypt

## Abstract

**Background:**

Anemia affects pediatric quality of life. It can cause hypoxia and loss of concentration. There are a lot of iron supplements used in treating pediatric CKD-induced anemia our study aimed to evaluate the effectiveness of oral liposomal iron in treating pediatric CKD anemia compared tointravenous iron dextran.

**Methods:**

This randomized, parallel study was conducted on 60 CKD pediatric patients. The patients were categorized into two groups. Group 1 comprises 30 pediatric patients administered oral liposomal iron 30 mg/day for 3 months. Group 2 comprises 30 pediatric patients administered intravenous iron dextran 50 mg three times/ week for 3 months.

**Results:**

Both groups have no statistically significant difference in Hb, RBCS, MCV, PLTs, RDW-CV, Cr, Hepcidin, Ferritin, Iron, and Transferrin levels after 3 months of treatment. Liposomal iron has higher efficacy than IV dextran after 3 months of treatment since there is a significant difference within the group between before and after treatment period at Hb, RBCS, HCT, MCV, RDW-CV, Hepcidin, Ferritin, Iron, TIBC, and Transferrin levels with no reported side effects during the study period.

**Conclusion:**

Oral liposomal iron has higher efficacy than IV dextran, which is the traditional CKD anemia treatment in pediatric with no reported side effects.

**Impact:**

- The key message of our article is to determine a safe, effective, easy to use, and less costly treatment of chronic kidney disease anemia in pediatrics.
- Our study will add to the existing literature a suggestion of an effective and safe treatment for pediatric chronic disorder, the traditional treatment has a lot of serious side effects.
- The impact of our study is to treat anemia resulted from chronic kidney disease in pediatrics with oral drug that has higher efficacy than the traditional injectable drug, this will lead to higher rate of anemia treatment due to higher rate of children compliance.

## Introduction

Any damage to the kidney can lead to chronic kidney disease (CKD).^1^ The estimation of glomerular filtration rate (eGFR) is often used to determine CKD patients by utilizing serum creatinine levels or cystatin C levels with different formulas. So, developing improved eGFR equations, such as the revised Schwartz (CKiD) formulas, is the standard method in eGFR calculation.^2^

The KDIGO categorizes CKD into five classifications based on the eGFR: G1: GFR before 90 ml/min/1.73 m^2^, G2: GFR ranging from 60 to 89 ml/min/1.73 m^2^, G3a: GFR ranging from 45 to 59 ml/min/1.73 m^2^, G3b: GFR ranging from 30 to 44 ml/min/1.73 m^2^, G4: GFR ranging from 15 to 29 ml/min/1.73 m^2^, and finally G5: GFR less than 15 ml/min/1.73 m^2^ this stage called ESRD.^3^

Deficiency of erythropoietin production and decrease in iron levels can lead to CKD-induced anemia. Iron deficiency in CKD may be due to the complete depletion of human iron stores which is known as absolute iron deficiency or the inability to use available iron stores which is called a relative deficiency or functional deficiency.^4^

Iron is one of the most critical elements for fetuses, infants, and child growth. Humans can store and recycle iron, so the content of iron inside the body is determined by the rate at which iron is absorbed from the diet. Iron homeostasis depends on the balance between the absorption of iron and its release from cells where it is store.^5,6^

Hormonal regulation through hepcidin controls iron absorption, storage, and distribution inside the human body.^5,6^ Hepcidin controls the iron fluxes into the bloodstream, the absorption of iron in the intestine, the transportation of iron, which is recycled from macrophages, and the stored iron release from hepatic cells. Hepcidin regulation is dependent on plasma iron concentration feedback and iron reserves.^5^

Although treatment of anemia in pediatrics mainly relies on orally administered iron dosage forms, oral iron can cause a lot of gastrointestinal tract GIT side effects such as nausea, vomiting, flatulence, abdominal pain, diarrhea, constipation, dyspepsia, metallic taste and black or tarry stools.^6^

Recently, new formulations of oral iron appear to introduce treatment with more efficacy and reduced toxicity. Liposomal preparations have a higher effectiveness and notably lower adverse effects. With such oral preparations, injectable iron is a second-choice treatment for specific patients.^5^

Liposomal iron is regarded as an important development in treating iron deficiency anemia (IDA) that does not improve with traditional oral iron supplements. It is an advanced form of oral iron supplement that contains a ferrous pyrophosphate core enclosed by a phospholipid membrane. Liposomes have unique biocompatibility and biodegradability features with low toxicity properties, making them effective drug carriers that can deliver different drugs to target organs. Liposomes carry iron and prevent contact with GI mucosa so that iron can be absorbed directly from the intestinal wall.^7,8^

Micronization of ferric pyrophosphate particles increases iron solubility due to the increase of the surface area of iron molecules and their dissolution rate. Microencapsulation prevents the breakdown of iron by enzymes in the oral cavity or stomach.^7,8^ Also, it prevents iron particles from interacting with alkaline pancreatic juice and acidic gastric secretions, bile acids, and free radical interactions. Liposomes are then absorbed in the small intestine to deliver iron particles without degradations. Then, macrophages uptake liposomes by endocytosis and transport them to hepatocytes via the lymphatic system.^7,8^ Lysosomal enzymes dissolve liposomes in hepatocytes, making iron available for utilization.^7,8^

High serum hepcidin levels reduce iron gastrointestinal absorption and decrease the effectiveness of oral iron preparations in patients with CKD. IV iron preparations are commonly employed to treat IDA in patients with CKD, particularly in those with renal failure undergoing dialysis and patients who are unable to tolerate oral medication.^9^

IV iron effectively increases hemoglobin and iron in CKD patients but has higher relative allergic reactions or hypotension, which reverses this advantage. Also, it carries a higher risk for infection, oxidative stress, cardiovascular disease, decrease in renal function, and excessive amounts of serum iron.^9^

Some experts suggest that children who have end-stage renal disease (ESRD) on hemodialysis and receive erythropoiesis-stimulating agents require ongoing treatment with regular low doses of intravenous (IV) iron.^10^ However, this carries more risk for frequent side effects.

The primary aim of this study was to investigate the efficacy and tolerability of oral liposomal iron and IV iron dextran in CKD-induced anemia in paediatrics because IV dextran is the standard treatment of CKD-induced anemia in pediatric patients on regular hemodialysis it is taken 3 times/ week during hemodialysis sessions on the dialysis machine. The second aim was to determine the most effective and applicable drug from both drugs to treat CKD anaemia in pediatric.

## Methods

### Study design

A randomized parallel study was conducted on 60 pediatric patients in the Pediatric Nephrology Department and Pediatrics Hemodialysis Unit at Menoufia University hospitals. The study was done in accordance with the ethical guidelines of the Helsinki Declaration of 1964 and its subsequent revisions. The study received approval from the Research Ethics Committee of Menoufia University and was registered on ClinicalTrials.gov. with number NCT05714176. Every participant was provided with information regarding the advantages and potential drawbacks of the study. All patients or their caregivers provided written informed consent. The investigation was conducted from February 2023 to March 2024. Pediatric patients who were 18 years old or younger with CKD stages 3–5 were included in the study. We exclude from the study patients with CKD who were more than 18 years old, pediatric patients with current bleeding, patients with malignancy, patients with anemia caused by a disease other than CKD, and patients who have received blood transfusions.

### Study population and participants

**Fig. 1:**
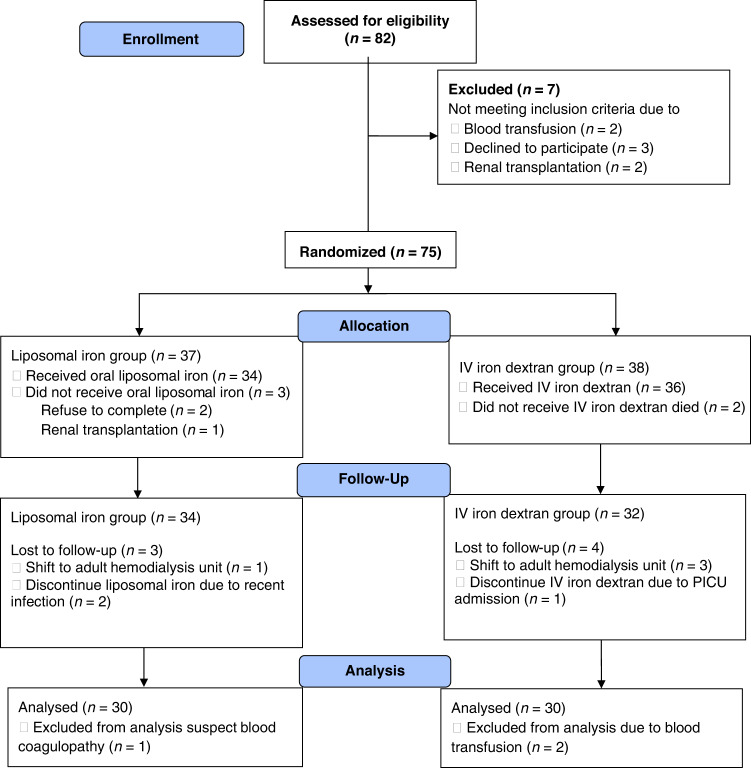
CONSORT flow diagram of the participant to illustrate the study progression during the controlled, parallel, randomized trial of liposomal iron group and IV iron dextran group.

The study was conducted on 60 pediatric patients. Patients were divided into 2 groups using a simple randomization method, as shown in CONSORT flow diagram (Fig. 1): group l:30 pediatric patients who received oral liposomal iron 30 mg/day for 3 months, and group 2: 30 pediatric patients who received IV iron dextran 50 mg/3 times weekly for 3 months.

### Sample size calculation

The sample size was determined using prior randomized (single-blind) non-inferiority clinical trial data. The study aimed to investigate the efficacy of liposomal iron in juvenile hemodialysis patients.^11^ Given an attrition rate of 10%, a total of 60 patients were recruited for our clinical trial, with each group consisting of 30 patients.

### Drug selection and dosing

IV dextran is a low molecular weight ferric hydroxide saccharate complex that is taken to all stage V pediatrics with CKD who are on regular hemodialysis 50 mg/3 times weekly during hemodialysis sessions on the dialysis machine to avoid drug administration side effects. The IV dextran dose was determined depending on the drug monograph, which mentioned that the hemodialysis pediatric dose is 50–100 mg/ dose. The liposomal iron dose was determined depending on manufacturing data, which noted that the dose is one ml/day, equal to 30 mg/day.

### Clinical end points

The primary clinical endpoint was the enhancement of oxygen-carrying capacity in patients’ blood, which will be evaluated in the two research groups by monitoring red blood cell count and hemoglobin level. The secondary endpoint was the alterations in serum concentrations of biological markers.

Blood parameters measured at the study’s start and after 3 months of treatment were Hb, RBCs, Platelets, Hematocrit, MCV, RDW-CV, and serum creatinine. Biological parameters were serum Ferritin, Hepecidin, serum Iron, Transferrin, and TIBC.

### Biochemical analysis

Blood samples collected from patients in all cohorts were assessed at the onset of the study period and again three months following therapy; the samples were collected predialysis in hemodialysis pediatrics. The samples were partitioned into two segments, one being utilized as a whole blood sample for analysis. Hb, RBCs, Platelets, Hematocrit, MCV, and RDW-CV using Hematology Analyzer BC-20s (Mindray® – Chinese) (Mindray Bc20s Hematology Reagent Diluent, China). Serum Iron and TIBC were assessed by colorimetric method using ARCHITECT ci4100 (Abbott®-United States).

The remaining portion was isolated using a centrifuge operating at 4000 revolutions per second in a medical laboratory electric desktop 80-1 low-speed centrifuge (CNWTC®, China) to get serum samples for the assessment of serum creatinine levels, Serum Ferritin, and Hepecidin the serum samples were stored at −18 °C for ELISA analysis. Serum creatinine was assessed by an automated chemical method using Clinchem multi-control level 1 mindray (Mindray® – Chinese) (Mindray Crea Reagent, China).

Serum Ferritin and hepcidin were assessed by enzyme-linked immunosorbent assay (ELISA) using PR 4100 Absorbance Microplate Reader (BIO RAD®, US). During ELISA analysis, pre-study and post-study samples were analyzed simultaneously to avoid batch effect, and samples were assessed in duplicates. Serum ferritin analysis was done using the ELISA kit SunRed company, China, Catalog Number 201-12-1703 96. Hepcidin analysis was done using the ELISA kit of Dldevelop company, China, with the catalog number DL-Hepc-Hu 96 Tests. Transferrin concentration is calculated from TIBC values.

### Statistical analysis

The data was organized and analyzed using Microsoft® Office Excel, 2019 (Microsoft Corporation). The statistical analysis was conducted using IBM-SPSS statistical package version 26.0 (IBM corporation software group, US). Numerical variables were expressed using mean, standard deviation, and range measures. A significance level of *P* < 0.05 (*) was used to determine a significant difference, while a significance level of *P* < 0.001 (**) was used to determine a highly significant difference. The normality test (The Shapiro-Wilk test) was used to examine the normality of the data. For parametric variables, an unpaired t-test was used to compare the two studied groups, and the paired t-test was used to compare the difference between before and after treatment in each group. For nonparametric variables, the Mann–Whitney test was used to compare the two groups at multiple comparisons, and the Wilcoxon test was used to compare the difference between before and after treatment in each group.

## Results

The demographic data for all participants in both groups are mentioned in Table 1.

**Table 1:** The demographic data of the participants.

Variable		Group 1 Liposomal iron	Group 2 IV dextran
		Mean ± SD	Mean ± SD
Age (Years)		10.23 ± 3.76	10.48 ± 3.51
Weight (Kg)		30.13 ± 11.54	27.26 ± 10.35
Height (cm)		121.33 ± 34.11	112.50 ± 40.97
BMI		23.73 ± 14.11	28.77 ± 19.46
Stage	4	3(10%)	0(0%)
	5	27(90%)	30(100%)
Gender	Male	23(76.7%)	21(70%)
	Female	7(23.3%)	9(30%)

Data are presented as mean ± SD, BMI: Body mass index

Group 1: 30 pediatric patients with CKD induced anemia administered oral liposomal iron 30 mg/day for 3 months.

Group 2: 30 pediatric patients with CKD induced anemia administered IV iron dextran 50 mg/3 times weekly for 3 months.

N.B All pediatric patients in both arms were on antihypertensive drugs (ACEIs or ARBs), vitamin D, calcium supplement, and iron supplement (IV iron dextran in all stage 5 pediatric patients and oral ferric hydroxide polymaltose in all stage 4 pediatric patients). During the study any other form of iron supplement was stopped.

Both groups have no statistically significant difference in Hb, RBCS, MCV, PLTs, RDW-CV, Cr, Hepcidin, Ferritin, Iron, and Transferrin levels after 3 months of treatment but have statistically significant differences in HCT and TIBC after 3 months of treatment as mentioned in Table 2.

**Table 2:** The change of the parameters of the participant before and after treatment.

Variable	Liposomal iron		IV iron dextran		*p* value
	Before	After	Before	After	
	Mean ± SD	Mean ± SD	Mean ± SD	Mean ± SD	
Hb (g/dl)	10.09 ± 1.38	11.68 ± 2.12	10.56 ± 1.58	11.17 ± 1.65	0.096
	0.001**		0.065		
RBCS (cellx10^6^/uL)	3.68 ± 0.70	4.22 ± 0.89	3.98 ± 0.69	4.07 ± 0.77	0.756
	0.001**		0.499		
PLTs (cell ×10^3^/uL)	272.33 ± 90.89	274.53 ± 107.43	319.03 ± 124.55	291.8 ± 162.43	0.135
	0.885		0.30*		
HCT (%)	31.16 ± 5.37	34.46 ± 7.41	33.27 ± 5.4	35.52 ± 4.94	0.504
	0.003*		0.051		
MCV (fL)	82.29 ± 7.02	85.02 ± 8.76	83.567 ± 5.56	85.17 ± 6.16	0.188
	0.006*		0.032*		
RDW-CV (%)	15.42 ± 1.26	16.61 ± 1.45	15.19 ± 1.33	16.31 ± 1.84	0.001**
	0.001**		0.002*		
Cr (mg/dl)	6.79 ± 2.37	7.37 ± 2.45	7.17 ± 2.19	7.72 ± 2.23	0.096
	0.105		0.115		
Hepecidin (pg/mL)	1357.52 ± 336.93	202.17 ± 127.01	801.17 ± 181.43	179.17 ± 99.48	0.756
	0.001**		0.001**		
Ferritin (ng/ml)	407.48 ± 421.37	241.14 ± 174.54	387.63 ± 344.64	398.8 ± 402.78	0.135
	0.002*		0.184		
Iron (ug/dl)	54.40 ± 24.92	78.39 ± 39.15	63.79 ± 16.56	80.66 ± 42.47	0.504
	0.001**		0.014*		
TIBC (ug/dl)	191.57 ± 82.05	252.3 ± 90.76	245.83 ± 48.64	286.87 ± 46.93	0.188
	0.001**		0.002*		
Transferrin (mg/dl)	166.07 ± 31.22	193.13 ± 39.02	178.73 ± 34.49	182.53 ± 43.05	0.001**
	0.001**		0.226		

Data are presented as mean** ± **SD, significant *P* < 0.05 (*) and highly significant *P* < 0.001 (**).

N.B Serum creatinine for hemodialysis patients was measured pre-dialysis.

*Hb* hemoglobin, *RBCs* red blood cells, *PLTs* platelets, *HCT* hematocrit, *MCV* mean corpuscular volume, *RDW-CV* red cell distribution width - coefficient of variation, *Cr* creatinine, *TIBC* Total iron binding capacity.

**Fig. 2:**
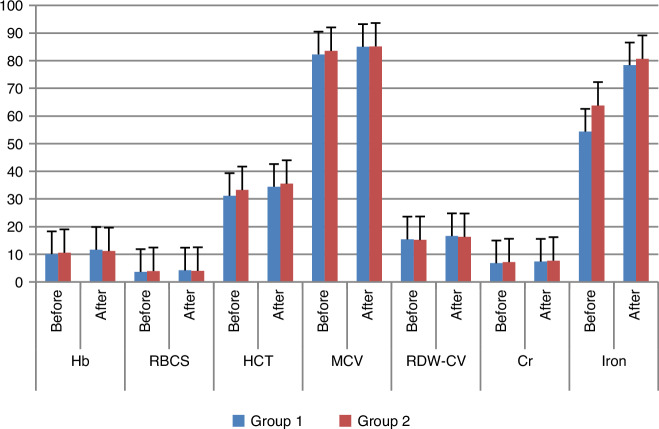
Group 1: 30 pediatric patients with CKD induced anemia administered oral liposomal iron 30 mg/day for 3 months. Group 2: 30 pediatric patients with CKD induced anemia administered IV iron dextran 50 mg/3 times weekly for 3 months. Hb hemoglobin, RBCs red blood cells, HCT hematocrit, MCV mean corpuscular volume, RDW-CV red cell distribution width - coefficient of variation, Cr creatinine.

**Fig. 3:**
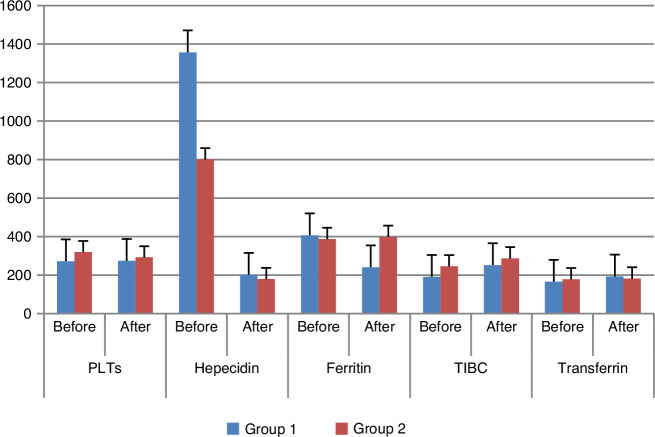
Group 1: 30 pediatric patients with CKD induced anemia administered oral liposomal iron 30 mg/day for 3 months. Group 2: 30 pediatric patients with CKD induced anemia administered IV iron dextran 50 mg/3 times weekly for 3 months. PLTs platelets, TIBC Total iron binding capacity.

Liposomal iron has higher efficacy than IV dextran after 3 months of treatment since there is a significant difference within the group between before and after treatment period at Hb, RBCS, HCT, MCV, RDW-CV, Hepcidin, Ferritin, Iron, TIBC, and Transferrin levels as shown in Figs. 2 and 3.

## Discussion

In this study, we studied the efficacy of oral liposomal iron in treatment of CKD induced anemia in pediatrics then compare its effect with the effect of IV iron dextran, which is the main treatment of IDA in CKD pediatric patients, especially pediatrics on regular dialysis.

The demographic data of all participants in both groups were collected and studied, as shown in Table 1.

Our results revealed no statistically significant differences between all studied parameters between the two groups after 3 months of treatment, except in HCT value and TIBC value the study period. Thus, oral liposomal iron has the same- efficacy of the mainstay treatment of this disease IV iron dextran or even it has better impact on the treatment of CKD-induced anemia. In addition, oral liposomal iron administration can avoid IV iron dextran side effects.

IV iron dextran can cause severe reactions, which can result from the drug itself or infusion. Its administration is more costly than oral liposomal iron.^12–14^ Also, IV preparations carry more risk of infection than any oral dosage form, especially in CKD pediatrics.^15^

During the study period, there were no reported side effects from pediatric patients or their caregiver administering oral liposomal iron 30 mg/day.

The results of our study indicate that oral liposomal iron significantly affects red blood cell count in CKD-induced anemia after 3 months of oral administration. There was a significant increase in Hb concentration (*p* = 0.001), RBCs count (*p* = 0.001), HCT (*p* = 0.003), MCV (*p* = 0.006), indicating a significant increase in RBCs average volume, and RDW-CV (*p* = 0.000) which means a highly significant difference in RBCs size which indicates the presence of anemia of chronic disorder.^16^ As shown in Table 2 and Fig. 2.

Although the administration of the conventional drug IV iron dextran didn’t produce significant effect on Hb concentration, RBCs count or HCT in CKD pediatrics who had anemia after 3 months of treatment (*p* = 0.065), (*p* = 0.499), and (*p* = 0.051) respectively. There was a significant increase in MCV (*p* = 0.032) and RDW-CV (*p* = 0.002) as mentioned in Table 2.

Our result agrees with the findings of another clinical study on children, which approved the idea that liposomal iron could significantly increase Hb levels.^17^

Oral liposomal iron had no significant effect on PLT count (*p* = 0.885), in contrast to IV dextran (*p* = 0.003), as shown in Fig. 3. This result means that IV dextran can increase the risk of thrombotic diseases in CKD pediatric patients who are at high risk of thrombocytosis and thrombotic disease due to CKD.^18^

IDA, in the case of CKD, can be absolute or functional. Absolute means decreased iron in the human body due to blood loss resulting from dialysis, GIT bleeding and decreased dietary iron intake.^12^ Functional iron deficiency is normal iron stores but decreased iron availability due to inflammation caused by increased hepcidin concentration.^12^

Our study showed a highly significant decrease in hepcidin concentration after 3 months of treatment in both groups (*p* = 0.001) as shown in Fig. 3.

Hepcidin is a hormone secreted by the liver in response to acute-phase reactions and plays a crucial role in maintaining the balance of iron in the body.^19,20^

The release of this substance is influenced by various variables, including iron levels, hypoxia, anemia, erythropoiesis, infection, and inflammation.^19^

Hepcidin attached to ferroportin (a protein transports iron through cell membrane) is responsible for the entry and degradation of iron into hepatocytes, enterocytes, and macrophages. Ferroportin prevents iron transport to plasma, leading to iron retention inside cells, decreased transferrin saturation, and reduction in the formation of new red blood cells. Inflammation increases hepcidin serum concentration, resulting in decreased intestinal iron absorption and release of stored iron, which leads to increased inflammatory serum iron concentration.^19^ A decrease in hepcidin can lead to an increase in ferroportin giving a better iron absorption.^19^ The decrease in hepcidin serum concentration in our study may be due to better iron hemostasis in the body resulting from better availability of iron used in new red blood cell formation, a decrease in the release of iron stores and an increase in ferroportin.

Results of our study revealed that liposomal iron caused a significant decrease in serum ferritin (*p* = 0.002), a highly significant increase in serum iron (*p* = 0.001), TIBC (*p* = 0.001), and transferrin (*p* = 0.001) after 3 months of treatment, which means liposomal iron can significantly increase circulating iron and iron stores in CKD pediatrics who are at increased risk of anemia due to absolute iron deficiency.

In CKD inflammation and oxidative stress cause a significant increase in ferritin and hepcidin serum levels independent from serum iron concentration. So, adequate iron intake may lead to decrease inflammation and oxidative stress that can lead to a decrease in ferritin and hepicidin serum levels.^21^

Conversely, IV dextran caused a significant increase in serum iron (*p *= 0.014) and TIBC (*p* = 0.002). Although their ‘wasn’t any significant change in serum ferritin (*p* = 0.184) and transferrin (*p* = 0.226), indicating high iron intake because iron is not excreted from the human body; it is recycled to be used inside the human body again.^22^

The higher effect of liposomal iron in increasing serum ferritin, serum iron, TIBC and transferrin levels may be due to the unique delivery mechanism, which penetrates the very constrictive typical gut walls, providing higher absorption and bioavailability results in decreased gastrointestinal side effects, higher plasma iron concentration, a rapid and efficient rise in Hb level leading to improved patient adherence and tolerance due to adaptable dosage options, cost-effectiveness, and better treatment outcomes.^13,23^

Our results are following many previous clinical studies that approved that liposomal iron can increase ferritin concentration and transferrin saturation in either normal or CKD pediatrics.^17,24^ Also, in a prior study on CKD patients who experienced gastrointestinal resistance to traditional oral iron treatment, oral liposomal iron was used instead for those patients. The hemoglobin serum level increased versus baseline and remained high 3 months after treatment. Adherence was 100%, and no patient reported adverse reactions to treatment. This result proves our study results that oral liposomal iron has higher efficacy in treating absolute iron deficiency due to poor iron absorption.^14^

There was no significant change in serum creatinine level in both group after 3 months of treatment. This indicates that both drugs haven’t any effect on renal functions.

From a financial point of view, liposomal iron is more costly than liposomal iron because only one bottle of liposomal iron can be used during the month. Still, the patient needs one ampoule every hemodialysis session, which is around 12 ampoule/ month.

The use of liposomal iron in treatment of CKD induced anemia in pediatrics has no serious risks. All pediatric patients don’t report any severe side effects during its administration. This fact is with accordance with results of another study that didn’t find any toxicity or serious side effects resulted from liposomal iron use in pediatrics.^24,25^ Although, as mentioned before IV iron dextran use in CKD pediatric patients carries significant risks of serious side effects (e.g., anaphylaxis, infusion reactions, or thromboemolism).^9,18^

There was a potential limitation of our study: the lack of previous research studies on our topic that led to determining the trial period according to patient’s compliance and determining doses based on manufacture labels. So, longitudinal studies need to be carried out for, more extended periods, and with higher doses to approve the drug efficacy and exclude anydrug-related side effects.

On the other hand, our study has many strengths, such as: (1) it is the first study conducted on oral liposomal iron comparison with the usual treatment IV iron dextran on CKD pediatrics. (2) The study population had nearly the same characteristics. (3) Randomization process was controlled and parallel. (4) Data processing method was the same for all participants.

## Conclusion

According to our study, oral liposomal iron has higher efficacy and effectiveness than the traditional IV dextran treatment in treating CKD-induced anemia in pediatrics. There ‘any reported side effects from oral liposomal iron during the study period. Oral liposomal iron introduces a new, easy, safe, and less costly way of treating anemia in CKD pediatrics with the same or even more efficacy of the mainstay traditional treatment IV dextran.

## Supplementary information

CONSORT-2010-Checklist

## Data Availability

The datasets generated during and/or analyzed during the current study are available from the corresponding author upon reasonable request.
